# Ultrasonic-Assisted Extraction of Astaxanthin Using Hydrophobic Deep Eutectic Solvent: Process Optimization and Anti-Aging Activity Evaluation

**DOI:** 10.3390/foods15122119

**Published:** 2026-06-12

**Authors:** Yuan Cao, Yalu Ji, Chong Chen, Wenyu Han, Zhijian Su

**Affiliations:** 1 School of Pharmaceutical Sciences, Wenzhou Medical University, Wenzhou 325000, China; cygrass1990@163.com; 2 Research and Development Department, Jilin Province Lanpu Haoye Technology Co., Ltd., Changchun 130051, China; 3 State Key Laboratory for Diagnosis and Treatment of Severe Zoonotic Infectious Diseases, Key Laboratory for Zoonosis Research of the Ministry of Education, Institute of Zoonosis, College of Veterinary Medicine, Jilin University, Changchun 130062, China; jiyalu120@163.com (Y.J.); m13944830520@163.com (C.C.); hanwy@jlu.edu.cn (W.H.); 4 Jiangsu Co-Innovation Center for the Prevention and Control of Important Animal Infectious Diseases and Zoonoses, Yangzhou University, Yangzhou 225009, China

**Keywords:** DL-menthol, acetic acid, Box–Behnken response surface methodology, process optimization, anti-aging activity

## Abstract

Deep eutectic solvent (DES) extraction is a green and efficient technology. As a substitute for organic reagents, DESs are widely used to extract active ingredients from traditional Chinese medicine. This study established an environmentally friendly and efficient method for extracting astaxanthin (AST) from *Phaffia rhodozyma* (PR) using ultrasound-assisted deep eutectic solvents (DESs-UAE). The astaxanthin content was determined by high-performance liquid chromatography (HPLC). Six types of deep eutectic solvents composed of DL-menthol and selected hydrogen bond donors were prepared and evaluated, among which the DL-menthol–acetic acid system showed superior extraction performance. Response surface methodology (RSM) was employed to optimize extraction parameters (ultrasonic power, time, and temperature), and the optimal conditions were determined as follows: ultrasonic power 420 W, ultrasonic time 20 min, and ultrasonic temperature 60 °C, achieving an AST extraction rate of 62% (2.49 mg/g). Compared with conventional organic solvent extraction, DESs exhibited a significantly higher AST extraction rate from PR, except for dimethyl sulfoxide (DMSO). Scanning electron microscopy (SEM) analysis demonstrated that DES-UAE treatment disrupted the cellular structure of PR, resulting in numerous surface pores; this facilitated the release of intracellular bioactive components and significantly improved AST extraction efficiency. The PR extract showed no significant cytotoxicity and could effectively promote L929 cell proliferation. It concentration-dependently increased superoxide dismutase (SOD) activity and decreased malondialdehyde (MDA) content in H_2_O_2_-induced oxidative stress L929 cells, thereby alleviating oxidative damage. Additionally, it concentration-dependently upregulated type I collagen expression in these cells, ameliorated the decline in collagen synthesis function, and exerted a protective effect against cellular oxidative damage. This study provides a green alternative to toxic solvents and offers important theoretical and chemical support for the extraction of natural products and the high-value utilization of *Phaffia rhodozyma* (PR). Deep eutectic solvents have emerged as promising green alternatives to hazardous organic solvents, yet hydrophobic DESs tailored for lipophilic astaxanthin extraction from *Phaffia rhodozyma* and the linkage between extraction performance and anti-aging bioactivity remain insufficiently explored. Here, an ultrasound-assisted hydrophobic deep eutectic solvent extraction strategy was constructed to acquire astaxanthin, aiming to overcome low efficiency and environmental risks of conventional organic extraction techniques. Six DL-menthol-based DESs were prepared and screened, and DL-menthol–acetic acid possessed the optimal extraction capacity. Key extraction parameters were optimized via response surface methodology, and the maximum astaxanthin extraction recovery reached 62% (2.49 mg/g) under 420 W ultrasonic power, 20 min treatment and 60 °C. This yield was markedly higher than that of most common organic solvents; though comparable extraction effect was obtained with DMSO, the adopted DES possessed outstanding low-toxic and biodegradable superiorities that DMSO cannot match. SEM characterization verified that the combined treatment destroyed yeast cell structure and formed porous morphology, which accelerated intracellular astaxanthin release and accounted for improved extraction efficiency. Biological assays proved the extract possessed good biosafety and proliferation-promoting effect on L929 cells. It effectively relieved cellular oxidative injury by elevating the SOD level and reducing MDA accumulation in oxidative damaged cells, and upregulated type I collagen expression to mitigate aging-related collagen loss. This work develops an eco-friendly and high-efficiency extraction route for lipophilic active substance, confirms the practical value of hydrophobic DES, and provides experimental basis for high-value utilization of *Phaffia rhodozyma* resources.

## 1. Introduction

Astaxanthin (AST), a naturally occurring lipophilic pigment belonging to the xanthophyll subclass of carotenoids [1], possesses multiple physiological activities, including antioxidant [2], anti-inflammatory [3,4], antitumor [5] and immunomodulatory effects [6]. These properties render it applicable in the pharmaceutical, food, dietary supplement, cosmetic and animal feed industries [7]. Natural astaxanthin exhibits potent antioxidant activity. By contrast, chemically synthesized astaxanthin has weak antioxidant capacity, accompanied by high production costs and residual intermediates and by-products; so, it cannot be directly used in human bodies [8]. Natural astaxanthin widely exists in *Phaffia rhodozyma*, microalgae, shrimps and crabs, and accumulates in birds via dietary intake [9,10,11].

**Figure 1:**
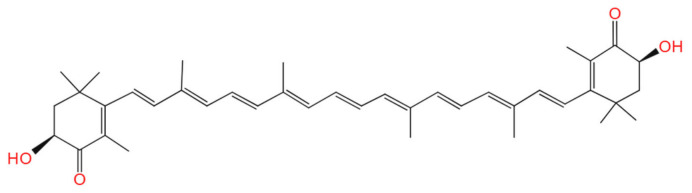
Chemical structure of AST.

As one of the major microbial sources of natural astaxanthin, *Phaffia rhodozyma* accumulates AST intracellularly. Its thick cell wall results in low bioavailability of this pigment [12]. Structurally, astaxanthin has a long conjugated double bond skeleton, which confers typical lipophilic characteristics. Due to this molecular feature, AST is barely soluble in water but shows good affinity for nonpolar and weakly polar solvents. This structural property fundamentally determines its dissolution and separation behaviors, laying an important theoretical foundation for the selection of customized DES extraction systems in this study (Figure 1). Traditional AST extraction commonly employs organic solvents [13,14], such as methanol, ethanol, n-butanol, ethyl acetate, acetone and dimethyl sulfoxide (DMSO). Nevertheless, these solvents pose environmental and operational safety hazards and tend to leave harmful residues [15]. Ultrasound-assisted extraction (UAE) can effectively improve extraction efficiency while reducing solvent consumption and energy loss, and has been widely applied as an auxiliary extraction technology.

Deep eutectic solvents (DESs) are low-melting liquid mixtures prepared from natural metabolites including choline derivatives, organic acids, amino acids, sugars and polyols, which serve as hydrogen bond donors (HBDs) and hydrogen bond acceptors (HBAs) at specific molar ratios [16,17]. DESs have outstanding advantages such as low cost, facile synthesis, abundant raw materials, environmental friendliness, low toxicity, high solvency and favorable chemical and thermal stability. Hence, they have become a research hotspot in green chemistry for extraction, catalysis and material preparation [18,19,20]. This type of solvent was first reported by Abbott et al. in 2004 [21]. Hydrophilic DESs can destroy hydration hydrogen bonds and induce bond dissociation in aqueous systems. Accordingly, hydrophobic DESs are particularly valuable for extraction processes involving aqueous media. For example, Pitacco et al. extracted astaxanthin from *Haematococcus pluvialis* using hydrophobic DESs and achieved excellent extraction efficiency [22]. Moreover, astaxanthin displays markedly higher solubility in acidic solutions than in neutral and alkaline media [23].

In this work, a natural eco-friendly DES combined with UAE was applied to extract AST from *Phaffia rhodozyma*. DESs have been widely acknowledged as green and efficient extractants for lignocellulose and biomass fractionation, serving as sustainable alternatives to traditional organic solvents in green extraction [24]. UAE is extensively utilized to enhance the extraction yield of bioactive compounds from natural resources [25]. We established an optimal extraction protocol by tuning key parameters, and clarified the extraction mechanism related to cell microstructure damage via scanning electron microscopy (SEM). Cell experiments verified that the obtained extract possessed strong antioxidant activity. This study not only develops an efficient and safe extraction strategy, but also provides scientific basis for high-value utilization and industrial exploitation of *Phaffia rhodozyma*. Previous studies, such as that of Mussagy et al. [26], mainly focused on single-factor optimization of astaxanthin extraction, generally adopting ionic liquids or conventional organic solvents as extractants. These traditional solvents usually suffer from poor biological safety and unsatisfactory biocompatibility.

In comparison, this study innovatively adopted a natural DES composed of DL-menthol and acetic acid as the green extraction medium. This system avoids the potential cytotoxicity and poor biodegradability of ionic liquids, and integrates the merits of environmental benignity and high extraction efficiency.

Beyond extraction parameter optimization, this study further breaks the limitations of previous research. The microscopic mechanism of cell damage induced by DES combined with ultrasound was revealed by SEM analysis. The Fourier-transform infrared spectroscopy (FTIR) results confirmed hydrogen bond formation inside DES molecules. Furthermore, the biological safety, antioxidative stress capacity and collagen synthesis regulation effect of the extract were systematically evaluated at the cellular level.

## 2. Materials and Methods

### 2.1. Chemicals and Reagents

This study utilized the following materials: natural AST (purity ≥ 96%, Beijing Solarbio Technology Co., Ltd., Beijing, China); PR (AST content approximately 0.4%, purchased from Weihai Dongxun Biological Technology Co., Ltd., Weihai, China); DMEM basic (1X) medium (Gibco, Thermo Fisher Scientific (China) Co., Ltd., Shanghai, China); BCA Protein Assay Kit (Beyotime Biotechnology Co., Ltd., Shanghai, China); Malondialdehyde (MDA) Assay Kit (Nanjing Jiancheng Bioengineering Institute, Nanjing, China); Type I Collagen ELISA Kit (eBioscience, Thermo Fisher Scientific (China) Co., Ltd.); Total Superoxide Dismutase (SOD) Assay Kit (Nanjing Jiancheng Bioengineering Institute); CCK-8 Cell Proliferation and Cytotoxicity Assay Kit (Solarbio Life Sciences Co., Ltd., Beijing, China); methanol (chromatographically pure, Thermo Fisher Scientific (China) Co., Ltd.); acetonitrile (chromatographically pure, Thermo Fisher Scientific (China) Co., Ltd.); phosphoric acid (analytically pure, Chengdu Kolan Chemical Co., Ltd., Chengdu, China); lactic acid (food-grade, Zhengzhou Kangyuan Chemical Products Co., Ltd., Zhengzhou, China); DL-menthol (analytically pure, Shanghai Macklin Biochemical Co., Ltd., Shanghai, China); and L929 murine fibroblast cells (obtained from the Chinese Academy of Sciences, Beijing, China).

The following instruments were used: AL204 Electric Heating Thermostatic Water Bath (Tianjin Taiste Instrument Co., Ltd., Tianjin, China); TGL-16 Centrifuge (Xiangyi Centrifuge Instrument Co., Ltd., Shanghai, China); AB135-S Analytical Balance (Mettler-Toledo International, Inc., Greifensee, Switzerland); JP300G Ultrasonic Extraction/Emulsification Unit (Wuhan Jiapeng Electronics Co., Ltd., Wuhan, China); 1260 Agilent High-Performance Liquid Chromatograph (Agilent Technologies, Inc., Santa Clara, CA, USA); and Epoch microplate reader (BioTek Instruments, Inc., Winooski, VT, USA).

### 2.2. DES Preparation

Six two-component hydrophobic DESs were prepared with slight modifications based on previously reported protocols, including DL-menthol–lactic acid, DL-menthol–nonanoic acid, DL-menthol–acetic acid, DL-menthol–propanoic acid, DL-menthol–valeric acid and DL-menthol–octanoic acid. The mixtures were blended at a specific molar ratio, heated at 70 °C and magnetically stirred at 500 rpm for 2 h until fully melted. Stirring was continued to obtain homogeneous and transparent liquid, and the prepared DESs were stored away from light at room temperature for subsequent use. DESs [27,28] were prepared according to the formula proportions in Table 1.

### 2.3. AST Content Determination

#### 2.3.1. Liquid Chromatography Conditions

The chromatographic column used in this experiment was Agilent TC-C_18_ (4.6 mm × 250 mm, 5 µm); acetonitrile (A) −0.1% phosphoric acid water solution (B) = 90:10 (*v*:*v*), isocratic elution; injection volume was 10 µL; flow rate was 1.0 mL/min; detection wavelength was 474 nm; and the column temperature was 25 °C.

#### 2.3.2. Preparation of AST Standard Solution

Accurately weigh an appropriate amount of AST reference standard, add acetonitrile–tetrahydrofuran = 1:1 to make a 0.53 mg/mL AST solution, and then dilute it with methanol 1000 times to make a 0.53 μg/mL AST reference solution.

#### 2.3.3. Extraction of Test Sample Solution Based on DES

Take an appropriate amount of PR, grind it thoroughly in a mortar, then weigh approximately 150 mg of the ground PR. Add 15 mL of DES, perform ultrasonic extraction for 30 min, then centrifuge the mixture at 5000 r/min for 10 min after sonication. Transfer 200 μL of the supernatant to a 10 mL volumetric flask, dilute to volume with methanol, mix well, filter through a 0.22 μm microporous membrane, and use the resulting solution as the HPLC test sample.

#### 2.3.4. Methodology

The calibration curves were generated to analyze the contents by plotting the peak area vs. concentration using six data points. The limits of detection (LOD) and the limits of quantification (LOQ) were identified with S/N (signal-to-noise) ratios of 3 and 10, accordingly.

The reproducibility and accuracy of the underlined assay procedure were confirmed through both intra- and inter-day variability. In case of intra-day precision, a sample solution along with a standard solution was evaluated six times on the same day. The inter-day precision was evaluated by analyzing the sample and standard solutions (as in intra-day) on three different days, followed by calculating the RSD values for both intra- and inter-day precisions of the five target macamides.

The test for stability was conducted by evaluating one extracted sample throughout 12 h up to 2 days. The RSD was then calculated for the evaluation of precision and reproducibility.

In the robustness test, one portion of the test solution was used to examine the influences of flow rate, pH value and column temperature. The results showed that slight variations in determination conditions barely affected the test outcomes, indicating good reliability of the method.

### 2.4. Extraction Process Optimization

#### 2.4.1. Single-Factor Experiment

According to the above extraction method, the following five factors were each individually examined for their impact on the yield of AST extraction: DES molar ratio (1:1, 1:2, 1:3, 1:4, 1:5), liquid–material ratio (1:50, 1:60, 1:80, 1:100, 1:133), ultrasonic power (100 W, 200 W, 300 W, 400 W, 500 W), ultrasonic temperature (30 °C, 40 °C, 50 °C, 60 °C, 70 °C), and ultrasonic time (10 min, 20 min, 30 min, 40 min, 50 min).

#### 2.4.2. RSM Experimental Design

Based on the single-factor experiments, a three-factor and three-level Box–Behnken response surface optimization experiment was conducted with ultrasonic power (A), ultrasonic time (B), and ultrasonic temperature (C) as independent variables, and AST extraction rate (Y) as the response variable. The factors and levels of the response surface experiment are shown in Table 2.

### 2.5. Comparison with Conventional Extraction Solvents

Currently, AST extraction mostly relies on traditional organic reagents. Therefore, in this experiment, the optimal extraction parameters obtained from the previous response surface methodology experiment were adopted. *Phaffia rhodozyma* was extracted using methanol, ethanol, n-butanol, ethyl acetate, acetone, and dimethyl sulfoxide (DMSO), respectively, and their extraction effects were compared with those of DES.

### 2.6. Scanning Electron Microscopy (SEM) Analysis

Untreated PR and the freeze-dried PR residue after DES-UVA treatment were selected as test samples. All samples were subjected to vacuum gold sputtering on the surface, and then their surface structures were examined by scanning electron microscopy with the detection parameters set as follows: accelerating voltage (EHT) of 20 KV and working distance (WD) of 27 mm.

Untreated raw *Phaffia rhodozyma* material, along with freeze-dried residues obtained from extractions under optimal conditions using DES, water, methanol and ethanol, was subjected to gold sputter coating under vacuum, and then analyzed via SEM at an accelerating voltage of 20 kV with a working distance of 27 mm.

### 2.7. FT-IR Analysis

Samples for FT-IR analysis were prepared as potassium bromide pellets. The materials analyzed included DL-menthol, acetic acid, and their 1:3 mixture. Each sample was mixed with KBr powder, thoroughly ground, and pressed into a pellet. Spectra were acquired at room temperature over a wavenumber range of 400–4000 cm^−1^ with a resolution of 4 cm^−1^ and 10 accumulated scans.

### 2.8. In Vitro Antioxidant Activity of PR Extract on L929 Cells

PR was extracted under optimal conditions, and the extract was purified by macroporous adsorption resin to obtain a dried PR extract. The dried powder was dissolved in complete Dulbecco’s Modified Eagle Medium (DMEM) to prepare a 500 μg/mL stock solution, which was sterilized through a 0.22 μm filter membrane and then diluted to working concentrations of 20, 40, 60, 80, 100, and 120 μmol/L. These working solutions were stored at 4 °C for later use. Meanwhile, a 1.0 mmol/L H_2_O_2_ induction solution was freshly prepared with serum-free DMEM medium for subsequent experiments.

#### 2.8.1. Evaluation of PR Extract on L929 Cell Proliferation

L929 cells in the logarithmic growth phase were digested with trypsin, resuspended in complete medium, and seeded into a 96-well plate at a density of 1 × 10^3^ cells per well in 100 μL of medium per well. In parallel, a DMSO control group was included to assess the solvent baseline effect. Specifically, L929 cells were treated with DMSO at concentrations corresponding to those present in the DMSO-extracted astaxanthin samples (final DMSO concentration ≤ 0.1%, *v*/*v*), as previous studies have reported that DMSO itself possesses intrinsic antioxidant activity that may confound cell-based assay results [29,30]. The experiment was divided into a blank group, a normal control group, a H_2_O_2_ model group, and a sample administration group. To the blank group, 100 μL complete medium without cells was added; the normal control group was cultured with normal L929 cells without any treatment; the model group was treated with H_2_O_2_ to induce oxidative damage; and the sample groups were incubated with PR extract at gradient concentrations.

After incubation at 37 °C in a 5% CO_2_ incubator for 24 h, 10 μL of Cell Counting Kit-8 (CCK-8) reagent was added to each well, followed by incubation for another 2–4 h. The absorbance at 450 nm was measured using a microplate reader. The cell viability was calculated using the formula: Cell viability (%) = [(Absorbance of drug-added wells) − (Absorbance of blank wells)] ÷ [(Absorbance of drug-free wells) − (Absorbance of blank wells)] × 100. Non-cytotoxic concentrations that promoted cell proliferation were selected to determine the concentration range for subsequent experiments.

#### 2.8.2. Effect of PR Extract on Antioxidant Indicators of Oxidatively Damaged L929 Cells

According to the manufacturer’s protocols of the malondialdehyde (MDA) and superoxide dismutase (SOD, WST-1 method) assay kits, absorbance at 532 nm was determined. The MDA content and SOD activity of each group were calculated according to the standard curve to evaluate the antioxidant effect of PR extract on H_2_O_2_-induced oxidative injury in L929 cells.

#### 2.8.3. Effect of PR Extract on Collagen Production in Oxidatively Damaged L929 Cells

According to the instructions of the Type I Collagen ELISA Kit, standards and cell supernatants were added to a 96-well plate, followed sequentially by capture antibody, detection antibody, and chromogenic solution. After incubation in the dark for color development, absorbance at 450 nm was measured. The concentration of type I collagen in the supernatant was calculated using the standard curve to analyze the effect of PR extract on cellular collagen production.

### 2.9. Statistical Analysis

The response surface experiment was designed using Design-Expert 13, and data analysis was performed using GraphPad Prism 9.0 software. For multi-group data, the normality and homogeneity of variances in each dataset were first verified (Shapiro–Wilk test for normality and Levene’s test for homogeneity of variances). Measurement data that conformed to a normal distribution and showed homogeneous variances were analyzed using one-way analysis of variance (ANOVA), while rank sum test was used for data that were not normally distributed or exhibited heterogeneous variances. The results were expressed as mean ± standard deviation ($\bar{x}$ ± s), and a significance level of *p* < 0.05 was considered statistically significant.

## 3. Results

### 3.1. Methodological Validation

**Figure 2:**
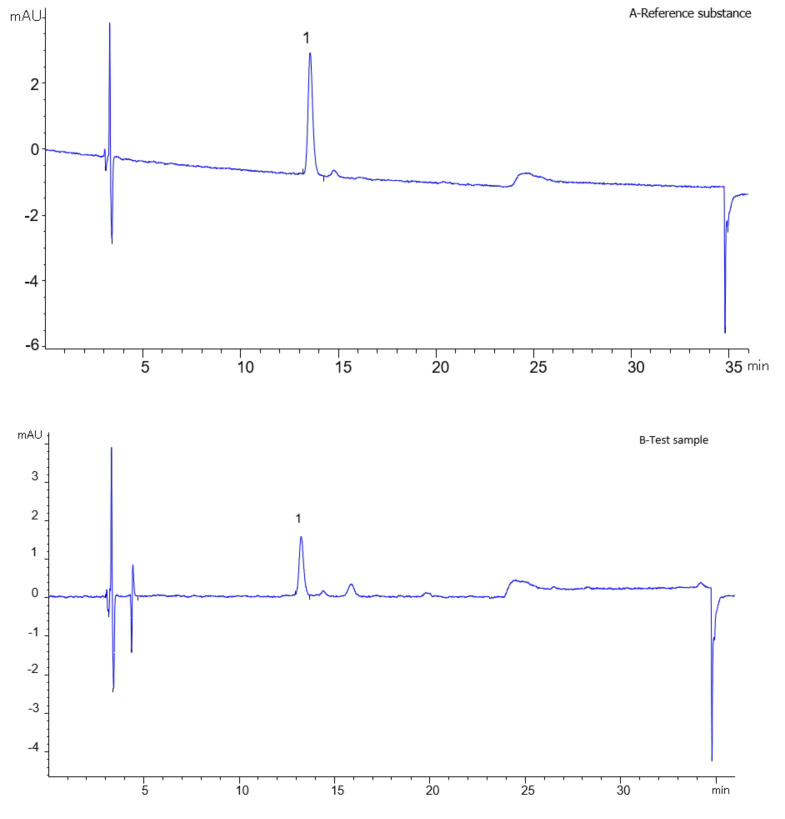
HPLC chromatograms (1. AST).

The control solution and test solution described in Section 2.3 were injected into the high-performance liquid chromatograph. The chromatographic analysis was conducted as per the method mentioned earlier, and the chromatogram obtained is shown in Figure 2. The separation of AST in the test component showed good results, and the theoretical plate number was greater than 3000.

Methodological validation was conducted for this analysis method. Six different concentrations of AST reference solutions were prepared and analyzed under the conditions specified above. The concentration of AST in the solutions was plotted on the *x*-axis, and the peak area on the *y*-axis for linear regression. The regression equation obtained was Y = 64.44X + 1.7117 (r = 0.9995), indicating a good linear relationship for AST concentration within the range of 0.13 to 2.65 μg/mL. The precision of this method was confirmed by analyzing the same AST reference solution six times, showing an RSD value of less than 2%. The repeatability of the test sample preparation method was also good, with an RSD value of less than 2% for the AST content in the same batch of PR extract solutions. Furthermore, the stability of these test sample solutions was confirmed by storing them at room temperature for 24 h and analyzing them at 4 h intervals; the RSD value of its AST peak area was less than 2%, indicating good stability.

The limit of quantification and limit of detection were 1.827 μg/mL and ≤0.548 μg/mL, respectively. The average recovery of astaxanthin ranged from 95.522% to 103.731%. Method durability was evaluated. Under different flow rates, the RSD values of astaxanthin retention time and peak area were 0.13–0.18% and 1.06–2.08%, respectively. When varying pH conditions, the RSDs of retention time and peak area were 0.10–0.61% and 0.81–1.78%. Under different temperatures, the RSDs of retention time and peak area were 0.09–0.45% and 1.25–2.45%.

### 3.2. Screening of DES

The solubility of AST in a solvent directly reflects its ability to extract AST, and increasing solubility is an effective means to improve the extraction rate [31]. The solvency of low eutectic solvents (DESs) depends on their structural composition. Generally, the viscosity of hydrophobic deep eutectic solvents is lower than that of hydrophilic solvents [32]. Cheng Wanting et al. [15] compared the solvency of AST in seven acidic DESs and found that they all outperformed ethanol. Among the tested systems, the DL-menthol–acetic acid solvent exhibited the best extraction efficiency, with an extraction yield of approximately 47% (1.27 mg/g). Therefore, based on this study’s results, we compared the extraction efficiency of the following six hydrophobic DESs compositions on AST in PR.

**Figure 3:**
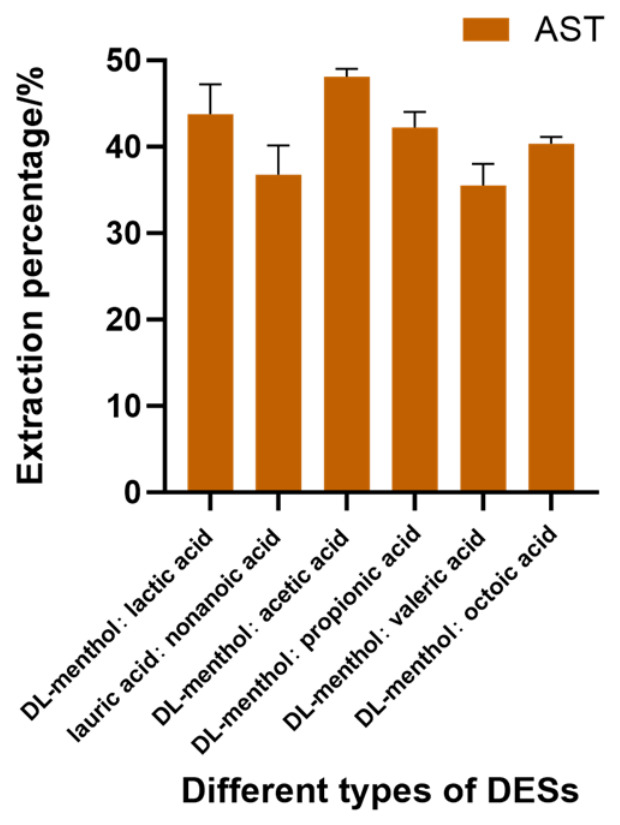
Effect of DES composition and molar ratio on AST extraction from PR.

A portion of PR was ground into a fine powder for later use. Approximately 150 mg of finely ground PR was weighed, and 15 mL of DES solvent was added for ultrasound-assisted extraction at a power of 250 W for 30 min and a temperature of 60 °C. After extraction, the solution was centrifuged at 5000 rpm for 10 min, and the supernatant was collected to determine the extraction rate of AST in PR. As shown in Figure 3, different compositions of DESs have a significant impact on the extraction rate of AST. When DES composition number 3, the DL-menthol–acetic acid combination, was used, the extraction rate of AST in PR was the highest. Therefore, we chose the DES composition with the highest AST extraction rate for further experiments.

### 3.3. Results of Single-Factor Experiments

**Figure 4:**
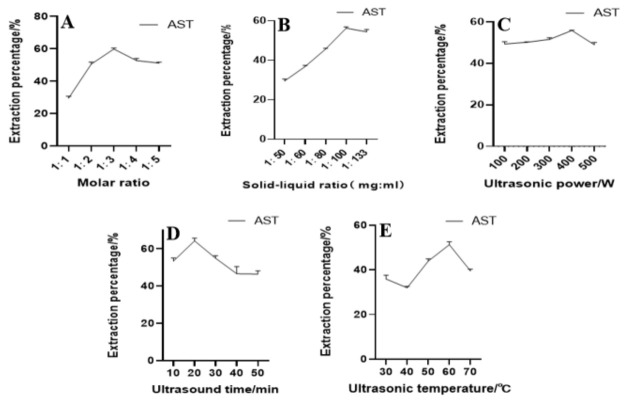
Single-factor investigation results of AST extraction process in PR (error bars represent the standard deviation of three parallel replicates n = 3) (**A**) Molar ratio; (**B**) Solid-Liquid ratio; (**C**) Ultrasonic power; (**D**) Ultrasound time; (**E**) Ultrasonic temperature.

After determining the DES composition, we continued to investigate the effect of molar ratios (1:1, 1:2, 1:3, 1:4, 1:5) on the extraction rate of AST in PR (Figure 4A). The molar ratio of hydrogen bond acceptor to hydrogen bond donor in DES is also an important factor affecting extraction efficiency [33,34]. When the molar ratio of DL-menthol–acetic acid was 1:3, the extraction rate of AST in PR reached its highest value. Therefore, the optimal DES composition was determined to be DL-menthol–acetic acid = 1:3 (molar ratio).

During extraction, the amount of solvent used is often positively correlated with the extraction rate. Sufficient solvent can help extract the active substances more thoroughly. Excessive extraction solvent may cause a dilution effect on target extracts [35]. About 150 mg of finely ground PR was taken, and DES-13 (molar ratio 1:3) solvent was added in different proportions for ultrasound-assisted extraction at a power of 250 W for 30 min, with an ultrasound extraction temperature of 60 °C. The changes in the extraction rate were investigated for different solid–liquid ratios (1:50, 1:60, 1:80, 1:100, 1:133). As shown in Figure 4B, with an increase in the solid–liquid ratio, the extraction rate of AST also increased. When the solid–liquid ratio reached 1:100, the extraction rate was the highest. Beyond a solid–liquid ratio of 1:100, the extraction rate no longer showed significant changes, indicating that the solvent amount had reached saturation. Therefore, all subsequent experiments were conducted using a solid–liquid ratio of 1:100 for extraction.

Ultrasound power directly affects extraction efficiency, but excessive power often damages the structure of active ingredients, leading to extraction failure [36]. Therefore, when selecting ultrasound-assisted extraction, it is important to consider choosing an appropriate ultrasound power. About 150 mg of finely ground PR was weighed, and 15 mL of DES-3 (molar ratio 1:3) was added. The ultrasound extraction time was 30 min, and the ultrasound extraction temperature was 60 °C. Ultrasound power levels of 100 W, 200 W, 300 W, 400 W, and 500 W were tested. According to the results in Figure 4C, an ultrasound power of 400 W yielded the highest extraction rate for AST, exceeding 60%. This indicates effective extraction of AST from PR.

As known, extraction time is one of the important factors determining extraction efficiency. Longer extraction times can lead to more thorough extraction of active components from the sample. However, after reaching a peak, extraction efficiency decreases [37,38]. This study used the extraction rate of AST in PR as an indicator and investigated the extraction efficiency at five different ultrasound extraction time points: 10 min, 20 min, 30 min, 40 min, and 50 min. Other extraction conditions included DES-3 (molar ratio 1:3), a solvent-to-feed ratio of 1:100, ultrasound extraction power of 250 W, and ultrasound extraction temperature of 60 °C. As shown in Figure 4D, the highest extraction rate for AST was observed at 20 min. As the ultrasound time increased, the extraction rate slightly decreased, indicating that an ultrasound time of around 20 min is most suitable.

Temperature can affect the properties of DES, thus influencing extraction efficiency. Higher temperatures result in lower density of low eutectic solvents (DESs), accelerated thermal motion of DES molecules, weakened intermolecular interactions, and reduced viscosity of DES. Lower solvent viscosity leads to lower surface tension, greater droplet formation capability, and easier penetration into narrow spaces [36,39,40]. Approximately 150 mg of finely ground PR was weighed, and 15 mL of DES-3 (molar ratio 1:3) was added. Ultrasound-assisted extraction was performed at a power of 250 W for 30 min. The extraction efficiency of AST was investigated at ultrasound extraction temperatures of 30 °C, 40 °C, 50 °C, 60 °C, and 70 °C. As shown in Figure 4E, the highest extraction rate for AST was observed at an ultrasound temperature of 60 °C. Therefore, the optimal ultrasound extraction temperature was set at 60 °C.

### 3.4. Results of Response Surface Model

Based on the single-factor experiments, it is evident that ultrasound power, ultrasound time, and ultrasound temperature have a significant impact on AST extraction rates. Consequently, with a fixed DES composition of LD-menthol–acetic acid (molar ratio 1:3), a solid–liquid ratio of 1:100, ultrasound power (A), ultrasound time (B), and ultrasound temperature (C) were set as independent variables. The AST extraction rate in PR served as the response variable (Y), and a response surface methodology was employed to optimize the process, leading to the selection of the optimal extraction conditions. The design and results of the response surface optimization experiments are outlined in Table 3.

**Figure 5:**
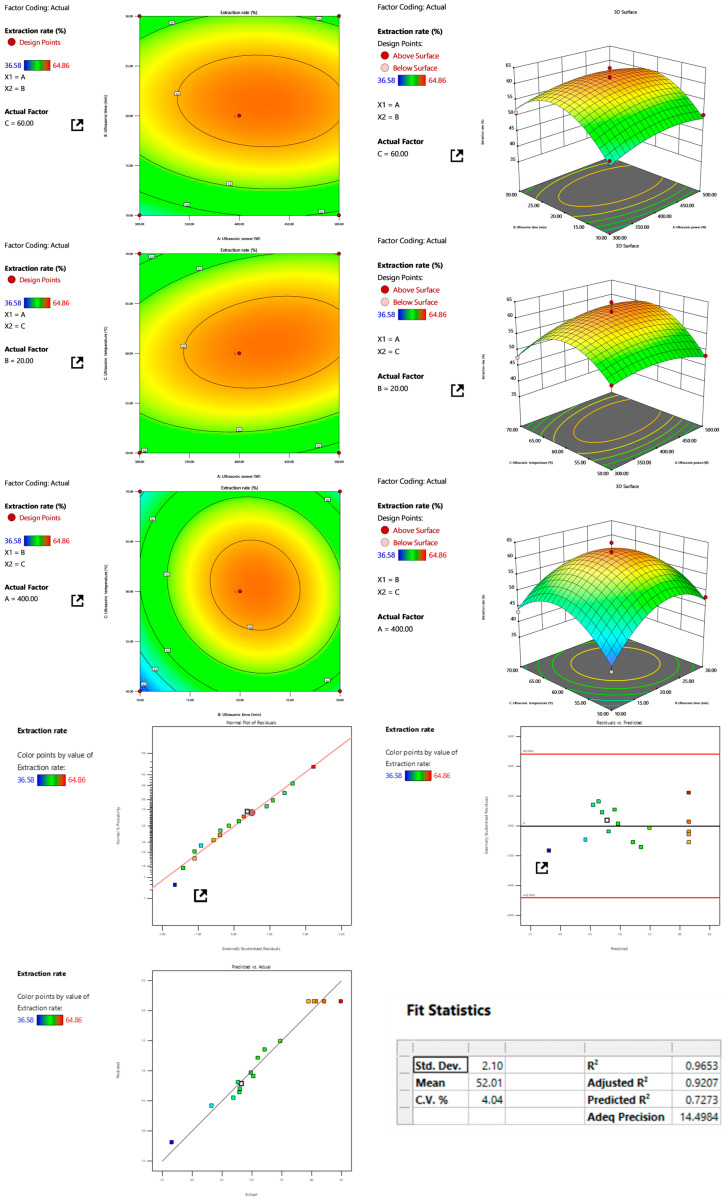
3D response surface and contour plots of factor interactions, together with model validation and goodness-of-fit statistics.

Analysis of the factor levels was performed using Design-Expert 13 software. This analysis resulted in a second-order polynomial regression model equation for AST extraction rate and a variance analysis of the response surface regression model. The 3D response surface plots and contour plots depicting the impact of ultrasound power (A), ultrasound time (B), and ultrasound temperature (C) on AST extraction in PR were generated (Figure 5).

The second-order polynomial regression equation is given as follows: Y= 61.52 + 1.24A + 2.76B + 1.37C − 0.56AB + 2.16AC − 1.67BC − 2.63A^2^ − 8.82B^2^ − 8.76C^2^. Based on the results of the model variance analysis presented in Table 4, it is evident that the fitted model is highly significant (with an F-value of 21.63 and a *p*-value of 0.0003). The lack of fit is not significant (with an F-value of 1.03 and a *p*-value of 0.4686), indicating that there is no significant discrepancy between the measured and predicted values. This implies a low experimental error, making this regression model a reliable predictor. The R-squared values for the model are 0.9653 (predictive) and 0.9207 (adjusted), with a difference of less than 0.1, suggesting reasonable agreement. The model’s signal-to-noise ratio is 4.04, which generally indicates a favorable model. Therefore, this model can be used for the optimization of AST extraction from PR.

Statistical analysis of the significance of each factor reveals the following order of significance: B > C > A, meaning that ultrasound time is more significant than ultrasound temperature, which is more significant than ultrasound power. The first-order factor B and second-order factors B^2^ and C^2^ are all highly significant (*p* < 0.01), while the second-order factor A^2^ is significant (*p* < 0.05). Figure 4 illustrates the interaction effects between factors. Through software analysis, the optimal process for extracting AST from PR was determined.

### 3.5. Validation of Optimal Extraction Conditions

The theoretically optimal process predicted by the Box–Behnken design was an ultrasound power of 426.19 W, an ultrasound time of 21.39 min, and an ultrasound temperature of 60.97 °C, resulting in a theoretical AST extraction rate of 61.94%. In line with the actual operational conditions, the final extraction process for AST from PR was optimized to an ultrasound power of 420 W, an ultrasound time of 20 min, and an ultrasound temperature of 60 °C. Three parallel experiments were conducted under these conditions, resulting in an average AST extraction rate of 62.08% (2.49 mg/g). These results confirm that the optimized extraction process exhibits good stability and high repeatability.

### 3.6. Results of Comparison with Conventional Extraction Solvents

**Figure 6:**
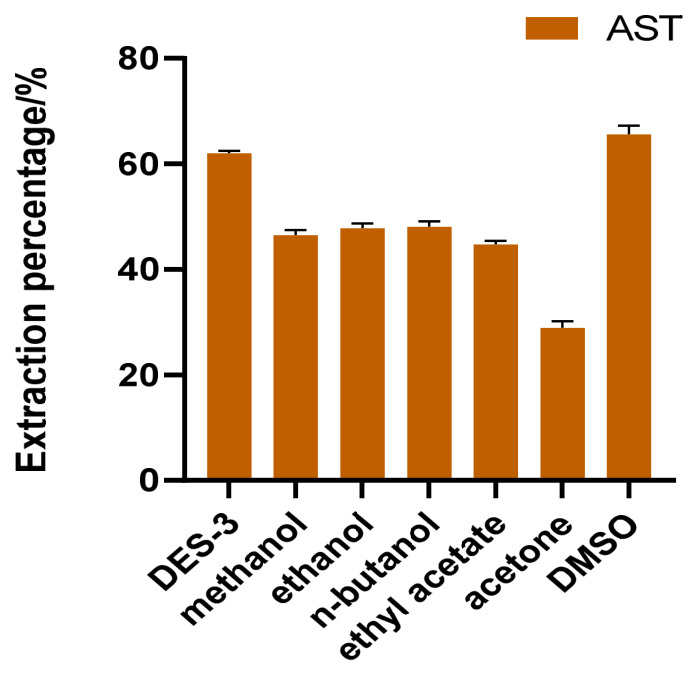
Comparison between DES and conventional solvents.

The samples obtained from the optimized extraction process were compared with samples extracted using traditional organic solvents. As indicated in Figure 6, DES, apart from DMSO, exhibited significantly higher extraction rates for AST from PR when compared to conventional organic solvents, with an improvement of up to 55.68%. Both DMSO and DES achieved extraction rates exceeding 60%, indicating that these methods are highly effective for extracting AST from PR. DMSO possesses relatively high toxicity, which tends to cause environmental pollution and harm human health.

### 3.7. Results of SEM Analysis

**Figure 7:**
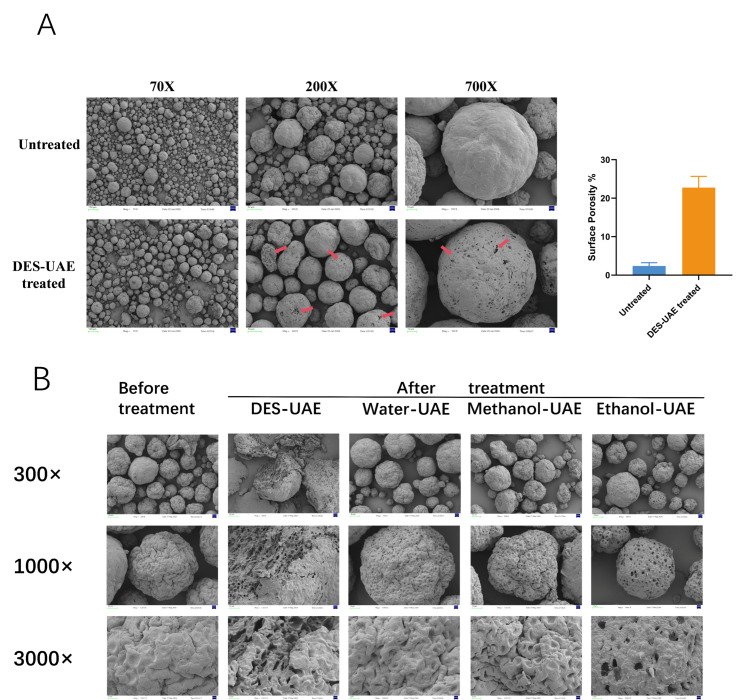
Results of SEM analysis ((**A**) SEM analysis of PR before and after treatment. (**B**) SEM analysis of *Phaffia rhodozyma* before and after treatment). The red arrows indicate the pores on the cell surface.

Figure 7 illustrates the morphological characteristics of PR (PR) before and after treatment, directly reflecting the effect of this treatment on the yeast cell surface structure. Untreated PR cells exhibited intact structures, dense morphology, and no obvious pores or damage on their surfaces. After treatment, the cell surface roughness increased significantly under 70× magnification, while numerous microporous structures were observed under higher magnifications of 200× and 700×. These results indicate that the combined treatment of grinding and DES-UAE can effectively disrupt the PR cell structure, promote the release of intracellular active components, and thus significantly improve the extraction efficiency of AST. Quantitative analysis via ImageJ 1.54p software demonstrated that the surface porosity of samples markedly increased from 2.38 ± 0.008% in the untreated group to 22.75 ± 2.906% in the deep eutectic solvent (DES)-treated group, which confirmed that DES could effectively destroy the superficial microstructure of samples.

The morphological changes in *Phaffia rhodozyma* cells before and after treatment with different solvents combined with UAE are shown in Figure 7B.

Untreated cells exhibited intact, smooth spherical structures without pores or fragmentation. In contrast, all solvent treatments caused varying degrees of structural disruption. Among them, DES-UAE induced the most severe damage: at 300× magnification, most cells were fragmented; at 1000× and 3000× magnifications, extensive honeycomb-like porous structures and deep cracks were observed, indicating complete destruction of cell wall integrity. Water–UAE and methanol–UAE only caused slight surface roughening with limited porosity, while ethanol–UAE led to moderate pore formation but retained the basic spherical morphology of the cells. The significant structural disruption by DES-UAE is directly correlated with its highest extraction yield, as such damage greatly facilitates the release of intracellular components.

### 3.8. Results of FT-IR Analysis

**Figure 8:**
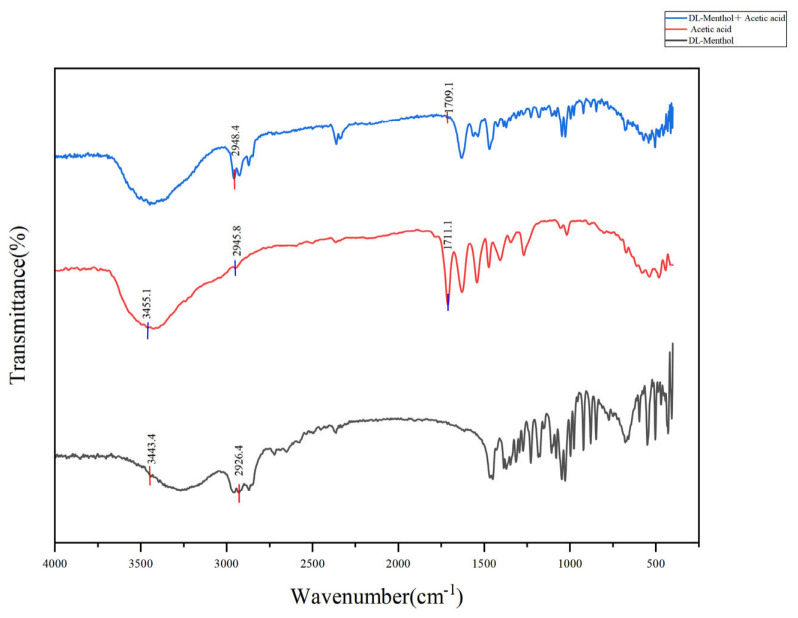
Infrared spectrograms of DES.

Fourier-transform infrared spectroscopy (FT-IR) was employed to characterize the functional groups of DL-menthol, acetic acid and their composite deep eutectic solvent (DES) based on the variations in molecular vibration characteristic absorption peaks so as to explore the intermolecular interactions between components (Figure 8). In the FT-IR spectrum of DL-menthol, the broad and strong characteristic peak at 3443.4 cm^−1^ was attributed to the stretching vibration of hydroxyl group (O–H). The absorption peak at 2926.4 cm^−1^ corresponded to the stretching vibration of saturated alkyl C–H bonds. For acetic acid, the broad intense peak at 3455.1 cm^−1^ originated from O–H stretching vibration, and the peak at 2945.8 cm^−1^ was assigned to saturated C–H stretching vibration. A strong absorption peak observed at 1711.1 cm^−1^ was the typical stretching vibration of the carbonyl group (C=O) in acetic acid. In the DES system, the C–H stretching vibration peak slightly shifted to 2948.4 cm^−1^. The original C=O stretching peak of acetic acid red-shifted from 1711.1 cm^−1^ to 1709.1 cm^−1^. Obvious red shifts in characteristic peaks were detected in the composite system compared with individual components, indicating a decreased electron cloud density around carbonyl and hydroxyl groups. This phenomenon demonstrated the formation of hydrogen bonds between DL-menthol and acetic acid. No significant changes were observed for major functional group absorption peaks of DES in the wavenumber range below 2000 cm^−1^, verifying that the molecular structure of the solvent remained stable during the extraction process [41,42].

### 3.9. In Vitro Antioxidant Activity of Xanthophyllomyces Dendrorhous Extract

#### 3.9.1. Evaluation of Cell Proliferation

**Figure 9:**
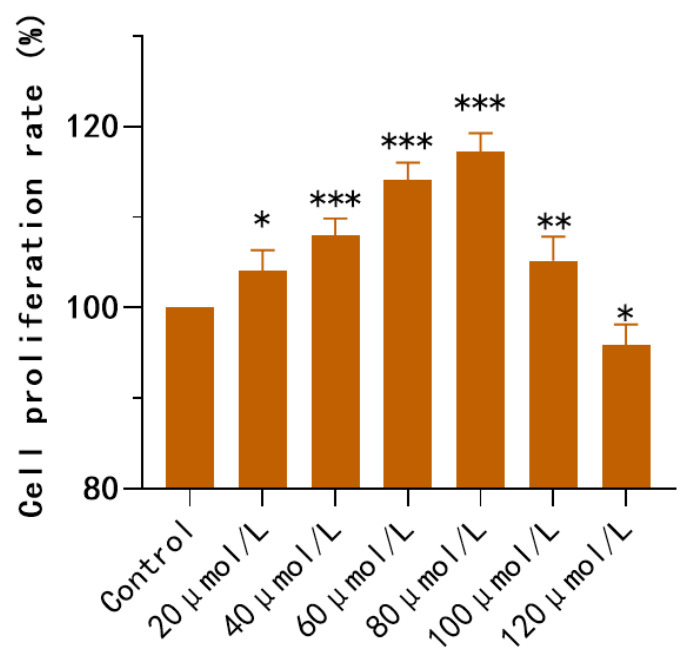
Effects of PR extract on the proliferation rate of L929 cells. Data are presented as mean ± SD, n = 3. * *p* < 0.05, ** *p* < 0.01, *** *p* < 0.001 versus the control group.

To evaluate the cytotoxicity and biological safety of PR extract, the CCK-8 assay was used to determine the proliferation rate of L929 murine epidermal fibroblasts (Figure 9). The results showed a concentration-dependent biphasic effect of the PR extract on cell proliferation: the cell proliferation rate remained above 100% at concentrations ranging from 20 to 100 μmol/L, with its proliferation-promoting effect increasing first and then decreasing with the rise in concentration, and peaking at 100 μmol/L (117.27%). Notably, the PR extract significantly promoted the proliferation of L929 cells at 40–80 μmol/L, indicating that the extract within this concentration range had no cytotoxicity but exhibited a proliferation-promoting effect. When the concentration increased to 120 μmol/L, the cell proliferation rate decreased slightly to 95.91%, suggesting only mild proliferation inhibition without obvious cytotoxicity.

#### 3.9.2. Effects of PR Extract on the Antioxidant Capacity of Senescent L929 Cells

**Figure 10:**
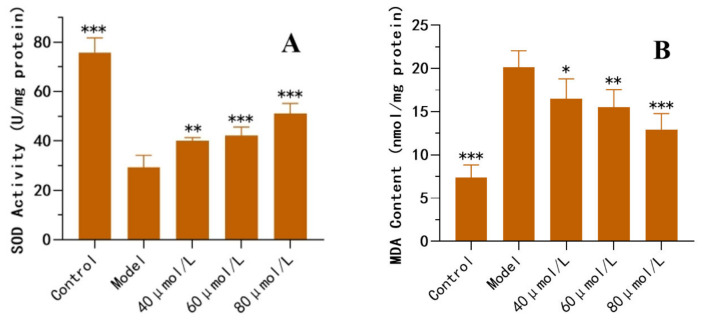
Effects of PR extract on SOD activity and MDA content in H_2_O_2_-induced L929 cells. Data are presented as mean ± SD, n = 3. * *p* < 0.05, ** *p* < 0.01, *** *p* < 0.001 versus the model group. (**A**) SOD activity assay; (**B**) MDA oxidative stress assay.

Superoxide dismutase (SOD) is a key intracellular antioxidant enzyme that maintains cellular redox homeostasis by scavenging reactive oxygen free radicals, and its activity level directly reflects the antioxidant capacity of cells. Malondialdehyde (MDA), as the end product of lipid peroxidation, is a classic biomarker for characterizing the degree of oxidative damage in organisms. As shown in Figure 10, an oxidative senescence model of L929 cells was established by H_2_O_2_ induction in this study, and the SOD activity of cells in the model group was extremely significantly decreased, while the MDA content was significantly increased. This indicated that the cellular redox homeostasis was disrupted, and the cells were in an obvious state of oxidative stress, confirming the successful establishment of the oxidative senescence model. After intervention with PR extract, the SOD activity of senescent L929 cells increased significantly, whereas the MDA content decreased markedly, which suggested that the cellular oxidative stress state was effectively alleviated. Moreover, the antioxidant effect of the extract exhibited a distinct dose dependence: the ameliorative effect was the most significant at the concentration of 80 μmol/L, followed by 60 μmol/L, and relatively weak at 40 μmol/L. The above results demonstrated that PR extract could effectively alleviate H_2_O_2_-induced cellular oxidative damage by enhancing intracellular SOD activity and reducing the production of MDA, a lipid peroxidation product. However, the indicator levels of all treatment groups had not yet recovered to the baseline levels of the blank control group. Collectively, these findings confirmed that PR extract exerted prominent antioxidant effects and effectively ameliorated oxidative damage in senescent L929 cells. Nevertheless, the upstream molecular regulatory network remains to be further clarified. In subsequent experiments, we will detect the expression levels of core proteins such as Nrf2 and Keap1 via Western blot assay so as to systematically elucidate the potential molecular mechanism by which PR extract mediates oxidative stress response. In addition, the antioxidant regulation of PR extract is closely associated with the canonical Nrf2/Keap1 oxidative stress signaling pathway [43]. Under oxidative stress, Keap1 binds to and promotes the degradation of Nrf2, impairing cellular antioxidant capacity and exacerbating oxidative damage [44]. PR extract effectively activates the Nrf2 pathway, facilitates Nrf2 nuclear translocation, and thereby upregulates the expression of downstream antioxidant enzymes including SOD. Increased SOD activity scavenges intracellular reactive oxygen species, mitigates lipid peroxidation, and reduces MDA accumulation. Consequently, PR extract alleviates H_2_O_2_-induced oxidative stress and delays cellular senescence via modulating the Nrf2-mediated antioxidant defense system.

#### 3.9.3. Regulation of PR Extract on Collagen Expression in Senescent L929 Cells

Fibroblasts are the primary cells responsible for collagen synthesis in the dermis, and the type I collagen they secrete is a key substance for maintaining the structural integrity and elasticity of the skin, as well as retarding the process of cellular senescence. The L929 cell senescence model induced by H_2_O_2_ can lead to a decline in cellular collagen synthesis capacity; thus, the variation in type I collagen content is an important indicator for investigating the anti-senescence effect of test substances.

**Figure 11:**
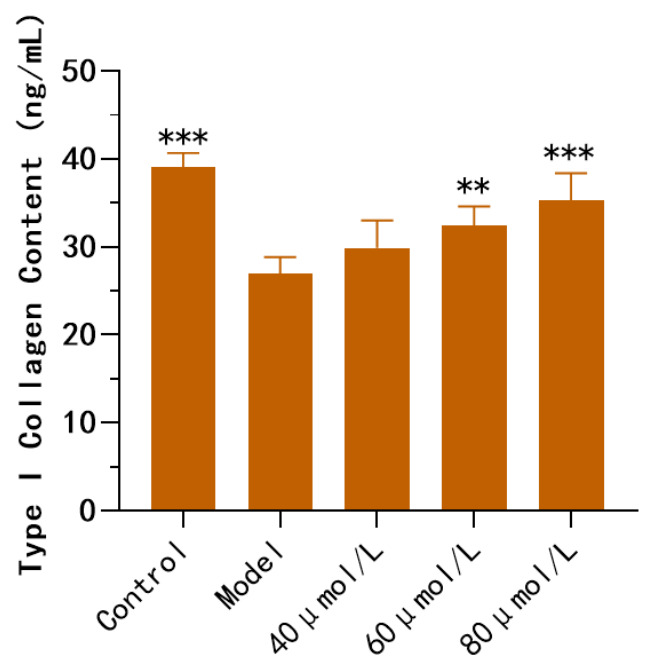
Effects of PR on the content of type I collagen in H_2_O_2_-induced L929 cells. Data are presented as mean ± SD, n = 3. ** *p* < 0.01, *** *p* < 0.001 versus the model group.

As shown in Figure 11, compared with the blank control group, the content of type I collagen in the model group was extremely significantly decreased, suggesting that H_2_O_2_ induction successfully caused the decline in collagen synthesis function associated with senescence in L929 cells. After treatment with different concentrations of PR extract, the content of type I collagen in all administration groups was significantly higher than that in the model group and increased in a concentration-dependent manner. Among them, the 80 μmol/L concentration group exhibited the most significant promotion effect, followed by the 60 μmol/L concentration group, and the 40 μmol/L concentration group also showed a notable improvement. In conclusion, PR extract can effectively upregulate the expression of type I collagen in H_2_O_2_-induced senescent L929 cells, ameliorate the functional decline in senescent cells, and thereby exert a role in retarding cellular senescence.

## 4. Discussion and Conclusions

As a novel class of green solvents, DESs possess unique merits, including facile preparation, low toxicity, biodegradability and outstanding solubility, and thus show promising prospects in the extraction of bioactive components from natural products. They are gradually replacing traditional organic solvents such as methanol and chloroform and becoming the core medium of green extraction technology [45,46]. Compared with hydrophilic deep eutectic solvents, hydrophobic deep eutectic solvents (HDESs) are more applicable to extract lipophilic active substances. They effectively solve compatibility problems between hydrophilic solvents and lipophilic targets, alleviate emulsification during extraction, and simplify subsequent separation and purification procedures [47]. In this study, HDESs were innovatively selected as extractants to efficiently obtain lipophilic AST from *Phaffia rhodozyma*. Combined with ultrasound-assisted extraction, an integrated green solvent intensified extraction process was constructed, offering a novel route for industrial eco-friendly production of AST.

Through solvent screening tests, DL-menthol–acetic acid (molar ratio 1:3) was determined as the optimal HDES for AST extraction in this work. The superiority of this system stems from the synergistic effect of multiple properties. Its short carbon chain structure matches the solubility characteristics of AST. Hydrogen bonding between acetic acid and DL-menthol, together with van der Waals forces among alkyl chains, forms a compact molecular stacking network. This structure increases system density and further boosts solubilization capacity toward AST. In addition, this HDES has far lower viscosity than ionic liquids and conventional DESs. Its favorable fluidity remarkably accelerates mass transfer, making it well-suited for practical extraction procedures [15,27]. Cassamo U. Mussagy et al. verified that DESs outperform choline-based ionic liquids in extracting AST from *Phaffia rhodozyma* [48]. This study further confirms that hydrophobicity and solubilization mechanism are critical determinants of extraction efficiency. The tunable molar ratio of DES allows for simultaneous extraction of AST and β-carotene. Moreover, the obtained extracts can be directly used without purification while maintaining biological activity.

Single-factor experiments determined the optimal process conditions as follows: a solid–liquid ratio of 1:100 (g/mL), ultrasonic power of 420 W, ultrasonic duration of 20 min, and extraction temperature of 60 °C. The variation in each parameter directly affects AST extraction efficiency. The solid–liquid ratio of 1:100 ensures adequate dissolution of AST in DES, avoiding incomplete extraction caused by insufficient solvent, and prevents resource waste and heavy subsequent treatment burden induced by excessive solvent. Ultrasonic compression–rarefaction cycles facilitate molecular movement. Acoustic cavitation destroys cell structure, modifies particle characteristics and accelerates mass transfer. It also raises tissue-swelling degree and promotes solute desorption and diffusion. All these effects jointly improve extraction performance. Nevertheless, ultrasonic power above 420 W generates excessive bubbles that block sound wave transmission. Meanwhile, accumulated heat cannot dissipate rapidly, triggering AST degradation and lowering extraction yield [49]. Extraction time and temperature both present a trend of moderate promotion but excessive inhibition. Within a reasonable range, extended extraction time enhances solute–solvent interaction and improves dissolution and mass transfer efficiency. Proper temperature elevation increases solvent diffusivity and solubility, thereby accelerating extraction. By contrast, overly prolonged time or excessively high temperature will degrade thermolabile active substances, resulting in reduced extraction efficiency [50,51]. On the basis of single-factor results, response surface methodology was applied to further optimize parameter interactions. The final AST extraction yield reached 62%. This value was remarkably higher than that obtained with most conventional organic solvents, and only slightly lower than that of DMSO extraction.

The SEM results directly prove that the combined treatment damages the cellular structure of *Phaffia rhodozyma*. This finding not only reveals the structural mechanism behind enhanced AST extraction efficiency, but also further validates the rationality and practicability of this extraction strategy.

AST is a natural high-efficiency antioxidant compound. *Phaffia rhodozyma* represents an important natural source of AST and possesses outstanding antioxidant potential owing to its abundant AST content [52]. Cell experiments in this study confirmed that the extract exhibited favorable biosafety and could markedly promote the proliferation of L929 cells. Its antioxidant activity was validated using the H_2_O_2_-induced oxidative senescence cell model. The extract effectively relieved cellular oxidative stress by elevating intracellular SOD activity and lowering MDA content, and this effect displayed obvious dose dependence, with optimal efficacy observed at 80 μmol/L. These findings provide direct molecular evidence supporting its antioxidant property and anti-senescence function. Previous studies on microbial physiological regulation have indicated that maintaining redox homeostasis and amino acid metabolism is critical for improving cellular stress resistance and antioxidant capacity [53]. Overactivation of oxidative stress pathways triggers massive intracellular ROS accumulation, which causes cell membrane lipid peroxidation and antioxidant system disorder, and ultimately leads to cellular oxidative injury. As a powerful antioxidant, AST modulates oxidative stress pathway activity in a concentration-dependent manner. Elevated AST content inhibits excessive pathway activation fundamentally via eliminating redundant ROS, boosting SOD activity and decreasing lipid peroxidation product MDA, thereby mitigating cellular oxidative damage. A negative regulatory correlation exists between AST concentration and oxidative stress response. Furthermore, the extract could upregulate type I collagen expression in senescent L929 cells dose-dependently and reverse the decrease in collagen synthesis induced by H_2_O_2_. It further proves that the extract can preserve skin structural integrity and retard skin aging via antioxidant action.

To further verify the superiority of the developed method, we conducted quantitative and qualitative comparisons between the DES–ultrasound-assisted extraction method and conventional organic solvent extraction in terms of recovery rate, time cost and practical applicability.

Conventional organic solvent extraction typically takes 60 to 120 min, with astaxanthin extraction recovery generally lower than 45%. Prolonged extraction time causes high energy consumption and raises the risk of active ingredient degradation. In addition, toxic organic solvents entail cumbersome post-processing procedures and potential safety risks.

In comparison, the optimal extraction time of our developed method is only 20 min, which greatly shortens the processing period and reduces energy consumption. The maximum astaxanthin extraction yield reaches 62% (2.49 mg/g), distinctly higher than those of most conventional extraction techniques. Meanwhile, the hydrophobic DES used has low toxicity and biodegradability, simplifying subsequent separation procedures and avoiding solvent residual pollution. Overall, this novel method realizes simultaneous improvements in extraction efficiency, recovery rate and environmental friendliness, and holds high practical application value.

Regarding the comparison with DMSO extraction, consistent with previous reports, DMSO exhibits the highest extraction efficiency among conventional organic solvents for astaxanthin. Our DES method achieved an extraction yield (62.08%) comparable to that of DMSO (62.5%, as shown in Figure 6). However, DMSO is known to possess intrinsic biological activities that can confound cell-based assay results. Several studies have reported that DMSO at concentrations above 0.5% (*v*/*v*) can induce cellular oxidative stress and affect cell viability, and even at lower concentrations (≤0.1%), DMSO may act as a free-radical scavenger, thereby interfering with antioxidant assessments. In contrast, the DL-menthol–acetic acid DES system used in this study exhibited no detectable cytotoxicity towards L929 cells within the tested concentration range (20–100 μmol/L) and even promoted cell proliferation. This superior safety profile, combined with comparable extraction efficiency, positions DES as a promising green alternative to DMSO for astaxanthin extraction in food and pharmaceutical applications.

In summary, this study established a DES-ultrasonic-assisted high-efficiency and green extraction process for AST, overcame the drawbacks of low efficiency and high pollution of traditional solvents, clarified the optimal extraction conditions, and provided theoretical and technical support for the application of DES in the extraction of fat-soluble natural products and the high-value utilization of PR. Compared with conventional organic solvent extraction, the developed DES-ultrasonic-assisted extraction system presents prominent green and sustainable merits. Common organic solvents such as methanol and chloroform are characterized by high toxicity, strong volatility and poor biodegradability, which easily cause volatile organic compound emission and residual contamination, accompanied by high energy consumption and massive waste liquid discharge during extraction, restricting their clean application in natural product separation [54,55]. By contrast, the DL-menthol–acetic acid-based natural DES adopted in this work possesses low toxicity, favorable biodegradability and extremely low volatility [56,57]. It can be simply prepared with high atom economy without hazardous byproduct generation [58]. Combined with ultrasonic assistance, the extraction period is shortened and energy consumption is reduced, further improving the environmental friendliness of the whole procedure. Consistent with mainstream green chemistry evaluation criteria, this strategy effectively avoids environmental and biosafety risks caused by traditional extractants, showing great potential for industrial green extraction of natural bioactive ingredients.

From the perspective of industrial scale-up and economic cost, the raw materials of DL-menthol and acetic acid are easily accessible with low market price, and the one-step mixing preparation of HDES requires no complex reaction equipment, which greatly reduces upfront production investment. Meanwhile, this hydrophobic DES can be recycled and reused via simple phase separation treatment after extraction, effectively cutting solvent consumption and production cost. Compared with traditional extraction technologies that demand lengthy procedures and large energy input, the ultrasonic-assisted extraction route adopted in this work has mild reaction conditions and compact technological flow, which is convenient for subsequent amplification and continuous production. Such advantages lay a solid foundation for its large-scale industrial promotion.

As a tunable green solvent, DES has no potential toxicity and broad application prospects. As a new generation of green extraction solvents, deep eutectic solvents feature low toxicity [45,59,60], biodegradability and stability, endowing them with broad application prospects [61].

## Figures and Tables

**Table 1:** DES composition and its molar ratio.

S/N	HBA	HBD	The Molar Ratio
1	DL-menthol	lactic acid	1:3
2	lauric acid	nonanoic acid	1:2
3	DL-menthol	acetic acid	1:2
4	DL-menthol	propionic acid	1:2
5	DL-menthol	valeric acid	1:2
6	DL-menthol	octoic acid	1:2

**Table 2:** Factors and levels of optimization of AST content by Box–Behnken test.

	Factor	A (Ultrasonic Power)/W	B (Ultrasonic Time)/min	C (Ultrasonic Temperature)/°C
Level				
−1		300	10	50
0		400	20	60
1		500	30	70

**Table 3:** Design and results of response surface methodology for optimization of AST extraction.

Run	Ultrasound Power (A)	Ultrasound Time (B)	Ultrasound Temperature (C)	Extraction Rate (Y)/%
1	500	20	50	48.26
2	500	10	60	50.24
3	300	10	60	46.89
4	400	20	60	59.49
5	400	10	50	36.58
6	300	30	60	51.02
7	500	30	60	52.13
8	400	20	60	60.40
9	400	30	70	47.95
10	400	10	70	43.25
11	400	20	60	64.86
12	300	20	50	49.84
13	300	20	70	47.68
14	500	20	70	54.75
15	400	20	60	60.74
16	400	20	60	62.09
17	400	30	50	47.98

**Table 4:** ANOVA for response surface quadratic model.

Source	Sum of Squares	df	Mean Square	F Value	*p*-Value	
Model	861.24	9	95.69	21.63	0.0003	significant
A	12.38	1	12.38	2.80	0.1383	
B	61.16	1	61.16	13.83	0.0075	**
C	15.04	1	15.04	3.40	0.1077	
AB	1.25	1	1.25	0.28	0.6108	
AC	18.71	1	18.71	4.23	0.0788	
BC	11.22	1	11.22	2.54	0.1552	
A^2^	29.05	1	29.05	6.57	0.0374	*
B^2^	327.49	1	327.49	74.03	<0.0001	***
C^2^	322.87	1	322.87	72.99	<0.0001	***
Residual	30.96	7	4.42			
Lack of Fit	13.50	3	4.50	1.03	0.4686	-
Pure Error	17.46	4	4.37			
Cor Total	892.20	16				

Note: “*” indicates *p* < 0.05; “**” indicates *p* < 0.01; “***” indicates *p* < 0.001; “-” indicates no significant difference (*p* > 0.05).

## Data Availability

The original contributions presented in this study are included in the article. Further inquiries can be directed to the corresponding author.
